# Zinc as a Biomarker of Cardiovascular Health

**DOI:** 10.3389/fnut.2021.686078

**Published:** 2021-07-30

**Authors:** Marija Knez, Maria Glibetic

**Affiliations:** Centre of Research Excellence in Nutrition and Metabolism, National Institute for Medical Research, University of Belgrade, Belgrade, Serbia

**Keywords:** zinc, cardiovascular diseases, zinc deficiency, zinc supplementation, obesity, hypertension, cardiovascular health

## Abstract

The importance of zinc (Zn) for cardiovascular health continuously gains recognition. As shown earlier, compromised Zn homeostasis and prolonged inflammation are common features in various cardiovascular diseases (CVDs). Similarly, Zn biochemistry alters several vascular processes, and Zn status is an important feature of cardiovascular health. Zn deficiency contributes to the development of CVDs; thus, Zn manipulations, including Zn supplementation, are beneficial for preventing and treating numerous cardiovascular (CV) disorders. Finally, additional long-term, well-designed studies, performed in various population groups, should be pursued to further clarify significant relationships between Zn and CVDs.

## Introduction

Zinc (Zn) is one of the most essential micronutrients involved in numerous crucial biological functions, i.e., cell differentiation and proliferation, cellular transport, DNA synthesis, endocrine, immune, and central nervous system functioning, reproduction, gene expression, and homeostasis (1). With the capacity to bind more than 300 enzymes and over 2,000 transcriptional factors, it is often regarded as a multipurpose trace element (2). Zn is a major antioxidant mineral responsible for inhibiting expansion and negative effects of free radicals and regulating the oxidant-antioxidant balance of cells (3). Zn deficiency significantly affects the functioning of biological systems, creates dysfunctions in humoral and cell-mediated immunity, consequently, increases the vulnerability to infections—predisposing people to disturbances in gut microbiota activity, increases the incidence of bacterial, viral, and fungal infections, and leads to the progression of chronic and degenerative diseases, i.e., type 2 diabetes mellitus (T2DM), cardiovascular diseases (CVDs), and cancers (3). CVDs are the leading cause of morbidity and mortality worldwide, and 17.9 million people died from CVDs in 2016, representing 31% of global deaths (4). CVD-related deaths are projected to reach 23.6 million annually by 2030 (1). Three-quarters of these deaths occur in low-income and middle-income countries (4). The deficiency of Zn affects 17% of the global population, up to 35% in low-income populations, i.e., South Asia and Africa (1). An association between Zn intake and Zn status with the pathogenesis of CVDs is demonstrated by several experimental and clinical studies (5, 6). Imbalances in Zn homeostasis contribute significantly to the development of CVDs, such as coronary heart disease (CHD), congestive heart failure (HF), ischemic cardiomyopathy (CM), myocardial infarction (MI), sudden cardiac death (SCD), and CVD mortality, in general (5). Antioxidant and prooxidant functions of Zn may have various positive effects on CV health and could prevent the development of CVDs (6).

This study provides a concise and thorough overview of the relationship between Zn homeostasis and CVDs. The importance and potential suitability of Zn status as a biomarker of CV health are discussed, highlighting present controversies and research gaps that entail further research studies.

## Zinc Deficiency—A Contributing Factor for Developing CVDs

Zinc is a major component of numerous enzymes within the human body. It controls the functioning of metalloenzymes, transcription factors, angiotensin-converting enzymes, desaturases, superoxide dismutases, and many others (1). Consequently, deficiency of Zn leads to apoptosis, inflammation, and oxidative stress, all well-acknowledged risk factors for the development of CVDs (7). Perturbations in Zn homeostasis affect the vascular endothelium (8). Zn deficiency weakens vascular health, impairs appropriate fatty acid and carbohydrate metabolism, and negatively impacts the cell structure of the aorta (9). Impaired Zn homeostasis is associated with common genomic and proteomic modifications that relate to CVDs (10). Zn controls the arteriosclerotic process, and inadequate Zn intake leads to increased oxidative stress, disrupted nitric oxide (NO), and nuclear factor kappa-light-chain-enhancer of activated B-cell (NF-kB) signaling and contributes considerably to endothelial damage and development of arteriosclerosis (ARS) (6). The rate of ARS, ischemic injuries, ischemic CM, and ischemic HF amplifies in line with decreasing plasma Zn levels (11, 12). Likewise, dietary Zn intake and Zn deficiency are adversely linked to subclinical ARS as demonstrated through carotid intima-media thickness (13, 14). Heart development is sensitive to Zn deficiency, and maternal Zn deficiency is linked to a high incidence of fetal heart abnormalities (15). Furthermore, Zn inadequacy prevents adequate development of cardiac tissues and increases blood pressure in fetuses and infants (16). Excessive embryonic cell death occurs after episodes of Zn deficiency (15). Proatherogenic factors, released during Zn deficiency, increase the incidence of arrhythmias, strokes, CM, and many other CV system pathologies (5, 17). There is an inverse relation between the serum Zn concentrations and the risk of CVDs in high-risk populations (18). Besides, lower serum Zn levels are associated with a higher risk of CVDs, with the greatest relations reported in most vulnerable populations, i.e., patients with diabetes and coronary angiography (18). Stimulated expression of inflammatory cytokines, i.e., interleukin 6 (IL-6), interleukin 2 (IL-2), interleukin 1 beta (IL-1β), tumor necrosis factor alpha (TNF-α), and increased oxidative stress are aggravated under Zn deficiency conditions (19). Similarly, cytokines can upregulate or downregulate the expression of particular cellular Zn transporters (20). Twenty-four Zn transporters are found within the human heart muscle tissue, so disturbances in Zn homeostasis may lead to CVDs (21). Turbulences in Zn homeostasis contribute to the development of hypertension (HT) (22). Through the renin-angiotensin-aldosterone system, Zn regulates arterial pressure and plays an important role in the etiopathogenesis of arterial HT (23).

Adequate Zn levels are a critical component in peroxisome proliferator-activated receptor signaling during atherosclerosis (ATS) (23). Furthermore, patients with coronary heart disease have poor Zn status (17). Zn deficiency contributes to the thickening of the vascular wall due to enhanced proliferation and hypertrophy (24). Low serum Zn levels are measured in people with HF (25, 26). Zn also has a role in redox signaling pathways, and it improves antiapoptotic, anti-inflammatory, and antioxidant activities (27). Deficiency of Zn can degenerate essential proteins like protein creatine kinase (C kinase), stimulate the production of inflammatory cytokines and C-reactive proteins, and may trap constituents in monocytes and macrophages (19). Serum Zn levels are considerably diminished in patients with left ventricular hypertrophy (LVH), and a significant inverse relation is seen between Zn status and LVH (5). Patients with ischemic stroke have lower serum Zn levels than healthy subjects (13). Similarly, lower serum Zn levels are seen in patients with HF and patients with left ventricular diastolic function (28). Besides, serum Zn levels are inversely associated with diminished glucose homeostasis and insulin resistance (29). Low serum Zn concentration predicts mortality in patients that need coronary angiography (30). What is more, serum Zn levels could be a valid diagnostic indicator for acute MI (31). According to the meta-analysis data, an increased prevalence of coronary artery disease (CAD) is linked to a lower dietary Zn intake, with a direct association between Zn status and MI (31).

## Zinc Interventions Alleviate Risk Factors for CVDs

Cohort studies, randomized trials, and meta-analyses of these studies propose that higher consumption of dietary Zn is linked to reduced risk of CVDs. Administration of Zn stimulates myocardial healing and improves arrhythmias (32). Besides, Zn is a wound-healing agent that supports cardiac steam cell survival, a critical element of cardiac healing (12). Zn supplementation has an atheroprotective effect (20) and contributes to a higher concentration of high-density lipoprotein cholesterol (HDL-C) and apoproteins, and lower total cholesterol (TC) levels (33). Higher serum Zn concentrations are associated with a decline in relative risk of death of CVDs (12). Reduced prevalence of CAD and T2DM is correlated with higher dietary Zn intakes (34, 35). Additionally, higher plasma Zn concentration is associated with a diminished risk of mortality of vascular disease (VD) (30, 36). Zn supplementation could potentially increase the effectiveness of currently used therapeutic drugs for managing CVDs (37). Finally, recently presented data of a systematic review and meta-analysis point out that low-dose and long-duration Zn interventions are of identical or in some instances of even larger magnitude and with even more beneficial effects compared to high-dose and short-duration interventions. Long-duration Zn studies, for 12 weeks or longer, alleviated risk factors for T2DM and CVDs, such as blood glucose, total fats, triglycerides (TGs), and low-density lipoprotein cholesterol (LDL-C), while the longer duration of low Zn doses affected a larger number of risk factors (38).

## Limited Knowledge on Vascular Roles of Zinc Transporter Proteins

Twenty-four Zip transporters are present within human heart muscle tissues, so disturbances in Zn homeostasis are strongly related to CVDs (21). Zrt, Irt-like protein2 (Zip2), Zip12, Zip14, and Zn transporter1 (ZnT1) and ZnT2 are linked to the vascular biology of CVDs (37).

For example, Zip2 has a beneficial role in the post-conditioning cardioprotective process (32). Zip2 polymorphism is associated with human carotid artery disease in the elderly (39). In addition, Zip12 is involved in the uptake of Zn into the vascular wall (22). Yet, limited information is available on vascular roles of Zip14, ZnT1, and ZnT4 (37).

Furthermore, ZnT1 is involved in cardiac electrophysiological effects of Zn and increased ZnT1 expression is seen in patients with atrial fibrillation (40, 41). Zn has a central role in the generation of NO and actions that have multiple implications for vascular endothelial and smooth muscle functions, i.e., vascular smooth muscle relaxation, antiplatelet properties, and protection of vascular endothelium against oxidative damage (42). The availability and function of NO are disturbed in Zn deficiency (37). The action of NO is controlled by both Zn and metallothionein (MT), so an insufficient supply of endothelial Zn will make NO ineffective as a CVD therapeutic agent (37).

Investigation of the genetic polymorphism of Zn transporters gains more and more attention. The polymorphism of Zn transporters confers a predisposition to various chronic and age-related diseases, such as chronic CVDs (43, 44). A common polymorphism in the ZnT8 gene, on the C allele, is associated with a higher risk of developing T2DM and metabolic syndrome (45, 46). Several single-nucleotide polymorphisms modulate Zn intake and status (47). There is an interaction between certain dietary components (i.e., omega three fatty acid intake) with Zn transporters in relation to the risk factors for CVD development (48). MT polymorphisms, MT1A, MT1B, MT2, and MT4, are often associated with dietetic neuropathy, blood pressure, inflammatory cytokine levels, DM, and CVDs (49–51). Similarly, there is an indirect involvement of uncoupling proteins in the MT-dependent reduction in the free radical-induced cardiac toxicity (52). Finally, ZnT1, ZnT4, ZnT5, ZnT6, ZnT7, and ZnT9 polymorphisms are linked to T2DM, dyslipidemia, and insulin resistance, all well-known CVD risk factors (53, 54).

## Zinc and Independent Risk Factors of CVDs

Several risk factors (i.e., T2DM, obesity, and HT) that predispose to VD are linked to irregularities in Zn homeostasis in individual organs or the whole body (55). A direct association between serum Zn and metabolic risk factors for the development of CVD, i.e., serum lipids, T2DM, and obesity, is shown (35, 55, 56). Zn plays an important role in insulin synthesis, crystallization, storage, and secretion in the pancreatic β-cells (57). Oxidative stress, a key risk factor in the pathogenesis of diabetes mellitus (DM), is aggravated under Zn deficiency states (58). Zn has insulin-mimicking properties, stimulates glucose uptake in insulin-dependent tissues, and regulates gluconeogenic enzymes (59).

ZnT8, located on dense core vesicles in β-cells, has a central role in the transportation of Zn into insulin secretory granules of β-cells and is identified as a novel therapeutic target in patients with diabetes (18, 60). Diabetes is often accompanied by hypozincemia and hyperzincuria (33, 61). Furthermore, Zn stimulates insulin binding to hepatocyte membranes and low Zn status considerably decreases the reaction of tissues to insulin (62). There is an inverse correlation between femur Zn and serum glucose concentrations (63).

Interestingly, a moderately high Zn intake could reduce the risk of diabetes by 13%, up to 40% in people living in rural areas (64). Zn supplementation improves glycemic control and reduces hemoglobin A1c (HbA1c) levels in patients with T2DM (63, 65). Besides, Zn improves glucose metabolism and contributes to glucose uptake into the relevant tissues (66). By inhibiting the activation of cytokines, Zn deficiency contributes to apoptosis and insulin resistance of β-pancreatic cells (57). The highest amount of Zn within the human body is stored in the pancreatic β-cells, so Zn ameliorates the consequences of immune-mediated free radicals in pancreatic islet cells (67). In addition, Zn stimulates phosphorylation of insulin receptor substrates and improves insulin sensitivity (68). Insulin resistance of adipocytes increases the release of fatty acids into the circulation and consequently improves fatty acid flux to the liver leading to hypertriglyceridemia (63). Similarly, Zn affects lipid metabolism directly. Zn maintains adipose tissue functioning *via* the activity of Zn finger proteins involved in the regulation of lipid metabolism (69). Zn-α2-glycoprotein inactivates hormone-sensitive lipase and accordingly reduces lipogenesis and increases lipolysis in adipose tissues (66). Zn modulates postprandial lipemia, and Zn deficiency markedly reduces the absorption rate of TGs, brings compositional alterations of chylomicrons, and reduces their production rates and uptake by the liver (70). Thus, Zn deficiency is often linked to obesity, due to chronic inflammation and oxidative stress. Zn levels in obese subjects are lower than in controls (71–73), and supplementation of Zn reduces plasma insulin resistance, leptin, and inflammatory biomarkers in obese individuals (74, 75).

## Zinc Homeostasis and CVDs—the Existing Controversies

The link between HT and Zn status is not decisive, and contradictory findings are reported over the years. Some studies demonstrate an inverse association (76, 77), while others found a direct positive link between serum Zn levels and blood pressure (57, 78). There are also data signifying no association between the two variables (74–81). Similarly, discrepancies in findings are reported for the risk of developing ARS in relation to serum Zn levels, and certain data reveal a direct link (6, 82), while others show no association between the serum Zn concentrations and ARS (83). The first randomized controlled trial (RCT) in humans shows adverse effects of Zn supplementation on HDL-C in healthy subjects (20). However, opposite findings exist, and a positive relation between serum Zn and HDL-C and LDL-C concentrations is observed (79). Lower consumption of dietary Zn is related to low HDL-C levels (31).

In addition, Zn supplementation has a beneficial effect on plasma lipid parameters, and it noticeably reduces TC, LDL-C, and TG levels in healthy individuals (10, 33). The benefits of Zn supplementation are more evident in non-healthy population groups. The meta-analysis data show that Zn supplementation leads to a significant reduction in LDL-C, TC, and TG levels in non-healthy patients, while in healthy people a noteworthy decline in TC levels is seen (33). HDL-C levels increase under Zn supplementation (20, 33, 77). Large longitudinal prospective cohort studies provide inconsistent findings on the association between supplementary Zn intake and risk of T2DM, showing both a direct, beneficial (34, 49, 83, 84), an inverse (85), and no relation (86, 87).

Likewise, there are no definitive conclusions on the relationship between Zn status and T2DM: no association (88) and an inverse link are reported (89) but, lower serum Zn levels are generally associated with an increased risk of T2DM (43, 90).

Different health status of participants, dissimilarities in the design, assessed outcomes across studies and influence of confounding factors and their appropriate adjustments, (i.e., medication, duration of the disease, dietary habits, and physical activity), differences in Zn assessment methods, lack of distinction in dietary Zn sources, variations in dietary data collection, and the inconsistency in utilized statistical models may all be potential reasons for observed discrepancies in findings among studies.

## Research Gaps and Recommendations for Further Research Studies

The precise role of Zn deficiency mechanisms in the pathogenesis of CVDs is still not known. The biological properties of Zn, playing a role in the physiology and pathology of CVDs, should be examined further. Additional community-based observational cohort studies may be useful for obtaining more precise and evidence-based conclusions on the relation between Zn and CVDs. It is essential to clarify the instances when inadequate dietary Zn intake and low Zn status are a result rather than a cause of CVDs. Particular attention should be paid to exclude the negative effects of medications of CVDs, i.e., diuretic furosemide, angiotensin receptor blockers, and angiotensin-converting enzyme inhibitors, on Zn status. Larger, well-designed randomized clinical trials are necessary to thoroughly examine the effect of Zn intake on CV health. Potential interactions with other dietary factors and micronutrients that could modulate Zn intake should be considered. Benefits, clinical applications, risks, and contraindications of dietary and supplemental Zn intake on main CV events should be examined further. The impact of the baseline Zn status on the efficacy of Zn interventions on CVD risk factors is of great importance and should be appropriately assessed and reported. Risk factors related to CVDs should be examined as primary outcomes of these interventions, and they should aim to examine the development and progression of these conditions. Further research studies should investigate the interaction between Zn intake and Zn status data with present preventative schemes and currently employed treatment methods that could help in the prevention and management of many ensuing CVDs.

As Zn status is affected by various factors, a careful selection of confounders should be made. Zn deficiency may not only be caused by an inappropriate dietary intake and/or bioavailability but also by factors such as age, physical activity, and alcohol or drug addictions. Further research studies should explore molecular mechanisms that support the sensing and distribution of Zn in various tissues. The interaction between Zn and inflammation deserves further research studies. The limitations of biomarkers of Zn status should be taken into consideration. Circulating plasma/serum Zn concentrations are affected by inflammation, time of the last meal, infections, and some other factors. All these elements have to be suitably deliberated. Newly proposed biomarkers of Zn status should be taken into consideration and investigated to CVD-related factors.

The mechanisms of action of Zn transporter proteins require additional research studies. Detailed and careful analysis of the activities of these transporters is required to improve our knowledge on the pathogenesis of CVDs. The transfer of information from Zn intake/status to cellular functions needs further extrapolation. New studies are needed to provide a more thorough understanding of MT and ZnT roles and the effects of their common genetic variations. Additional studies are required to explain the interactions between specific genetic profiles and zinc status. Further research studies should clarify gene-nutrient interactions and their relationship with Zn status and CVDs. It would be beneficial to develop suitable methods for measuring endothelial Zn as a biomarker of vascular Zn deficiency. The interplay between Zn and NO levels should be further investigated. The expression and functions/dysfunctions of Zn transporters in vascular tissues and genetic risk factors associated with Zn transporters should be additionally tested. Zn homeostasis is altered early in CVDs, so an intervention with Zn-related therapy could provide significant benefits. Preventative CVD actions should include programmed Zn nutrition approaches. The possibility of therapeutic manipulations of CVDs by Zn-based treatments exists; however, further low-dose short- and/or long-duration well-designed studies, across a variety of populations, are needed. The role of Zn supplementation in the process of recovery from CVDs should be more intensively investigated to find safe and desirable levels of Zn supplementation and, additionally, to determine the dose and duration that would be most beneficial primarily for the prevention of and, if need be, for the treatment of various ensuing CVD-related pathologies. Appropriate dietary recommendations, food fortification, and agronomic biofortification strategies should all be investigated and employed so that majority of people, both in developing and developed countries, can attain sufficient levels of dietary Zn in daily diets and potentially diminish the risk of developing CVDs.

## Author Contributions

MK conceptualized and wrote the manuscript and prepared the manuscript for submission. MG revised the final version of the manuscript. Both authors contributed to the article and approved the submitted version.

## Conflict of Interest

The authors declare that the research was conducted in the absence of any commercial or financial relationships that could be construed as a potential conflict of interest. The handling editor disclosed a past co-authorship with the authors MG and MK.

## Publisher's Note

All claims expressed in this article are solely those of the authors and do not necessarily represent those of their affiliated organizations, or those of the publisher, the editors and the reviewers. Any product that may be evaluated in this article, or claim that may be made by its manufacturer, is not guaranteed or endorsed by the publisher.
